# Linking Phytochemical Diversity to Aflatoxin Suppression: LC-MS/MS Metabolomics of *Trichilia dregeana* Bark Extracts

**DOI:** 10.3390/molecules31030578

**Published:** 2026-02-06

**Authors:** Martha Cebile Jobe, Babra Moyo, Ntakadzeni Edwin Madala, Mulunda Mwanza

**Affiliations:** 1 Food Security and Safety Niche Area, Faculty of Natural and Agricultural Sciences, North-West University, Mahikeng Campus, Mmabatho 2735, South Africa; 2 Department of Food Science and Technology, University of Venda, University Rd., Thohoyandou 0950, South Africa; barbara.moyo@univen.ac.za; 3 Center of Excellence in Mass Spectrometry for Southern Africa (CEMMSA), University of Venda, University Rd., Thohoyandou 0950, South Africa; ntakadzeni.madala@univen.ac.za; 4 Department of Animal Health, North-West University, Mahikeng Campus, Mmabatho 2735, South Africa

**Keywords:** *Trichilia dregeana*, LC-MS/MS metabolomics, *Aspergillus flavus*, aflatoxin, cereal grains

## Abstract

*Trichilia dregeana* has a rich phytochemical diversity and biological activity; however, information on its metabolomic profile and antimycotoxigenic potential is limited. This study investigated different extracts of *T. dregeana* bark obtained with various solvents (water, ethanol, ethyl acetate, and methanol), assessing their chemical composition using LC-MS and their inhibitory activity against the aflatoxin produced by *Aspergillus* fungi. LC-MS analysis identified metabolites belonging to several secondary metabolite classes, including flavonoids, phenolic acids, lignan glycosides, cardiac glycosides, coumarins, cinnamic acids, and limonoids. Solvent polarity strongly influenced metabolite distribution, with water and methanol enriching polar antioxidant compounds, while ethanol and ethyl acetate extracted semipolar antimicrobial constituents. The antimycotoxigenic efficacy of *T. dregeana* bark extracts was evaluated against *Aspergillus flavus* in maize, rice, and flour matrices. Among the tested extracts, only the methanolic extract exhibited a statistically significant reduction in aflatoxin levels (µg/kg), while the water, ethanol, and ethyl acetate extracts showed no significant inhibition. Fungal inoculation significantly increased aflatoxin levels, with maize showing the highest contamination (673.32 µg/kg). At 50 µg/mL extract, aflatoxin concentrations were reduced to 230.39 µg/kg maize, 129.93 µg/kg rice, and 143.89 µg/kg flour, with efficacy comparable to or exceeding the commercial fungicide tenazole. Associations between solvent-dependent metabolite class distribution and aflatoxin suppression were observed; however, bioactivity was demonstrated exclusively at the crude extract level. These findings suggest that methanolic extracts of *T. dregeana* bark may represent a promising natural alternative to antimycotoxin agents, warranting further fractionation and mechanistic validation.

## 1. Introduction

The safety and quality of animal feed are critical to global livestock productivity, food security, and public health. Contamination by pathogenic bacteria such as *Escherichia coli*, *Salmonella enterica*, and *Staphylococcus aureus*, along with toxigenic fungi such as *Aspergillus* and *Fusarium*, remains a major challenge [1,2]. Such microorganisms not only reduce feed nutritive value but also produce harmful mycotoxins, including aflatoxins and fumonisins, which pose risks to both animal and human health [3]. Concurrently, fungal spoilage and mycotoxin contamination impose substantial economic losses and food-chain risks. *Aspergillus* species, particularly aflatoxin-producing strains such as *A. flavus*, are among the most prevalent fungal contaminants of animal feed and pose a significant risk to feed safety due to their ability to produce aflatoxins [4,5].

Antibiotics and synthetic preservatives have been used as feed additives to control microbial damage and improve growth performance. However, the emergence of antimicrobial resistance and increased consumer demand for antibiotic-free livestock production necessitate the development of sustainable alternative agents [6]. Among the promising alternatives are phytogenic feed additives derived from medicinal plants, which are rich in bioactive compounds such as terpenoids, phenolics, alkaloids, and saponins [7,8]. Phytochemicals, especially terpenoids, phenolics, and alkaloids, offer multi-target antimicrobial actions and can be formulated as extracts, essential oils, or standardized phytogenic feed additives [9]. The Meliaceae family (*Azadirachta indica*, *Trichilia* spp.) is known for limonoids that function as insect antifeedants and display antibacterial and antifungal activities [10].

*Trichilia dregeana* is indigenous to southern and eastern Africa and is commonly known as the forest mahogany. Ethnobotanical records indicate its use in traditional medicine for treating infections, inflammation, and gastrointestinal disorders. Traditional uses include remedies for infectious and inflammatory conditions, suggesting bioactivity aligned with antimicrobial indications. In this study, we focus on its suitability as an antimicrobial agent in cereal grains. Preliminary studies have shown that extracts of *T. dregeana* exhibit antibacterial activity against *E. coli* and *S. aureus* [11]. Although direct antifungal data remain limited, analogous limonoids from related *Trichilia* species have demonstrated inhibitory effects against phytopathogenic fungi [12,13]. Given the urgent need for natural alternatives to antibiotics in feed, and the phytochemical richness of *T. dregeana*, systematic evaluation of this plant as a potential antimicrobial feed additive is required. Therefore, this study aimed to investigate the efficiency of different solvents (water, ethanol, ethyl acetate, and methanol) in extracting essential compounds (metabolites) from *T. dregeana* bark. Furthermore, the antimicrobial potential in cereal-based feed safety management was also investigated. Although several plant-derived extracts have demonstrated antifungal or antimycotoxigenic potential, the distinction between fungal growth inhibition and specific suppression of aflatoxin biosynthesis remains insufficiently explored. Moreover, limited information is available on how solvent-dependent extraction influences the phytochemical composition of *T. dregeana* bark and its antimycotoxigenic activity. This study hypothesizes that solvent-dependent extraction of phytochemicals from *T. dregeana* bark results in reduced antimycotoxigenic activity against *A. flavus*, which is attributed to antifungal growth inhibition that focuses on reducing the overall growth of fungi, thus suppressing aflatoxin biosynthesis.

## 2. Results

### 2.1. Metabolomic Profiling of T. dregeana Bark Extracts

Untargeted LC-MS/MS analysis identified a substantial number of metabolites across the water, ethanol, ethyl acetate, and methanol extracts of *T. dregeana* bark (Table 1). All compound identifications are reported as putative annotations (MSI level 2), based on accurate mass measurements, MS/MS fragmentation patterns, and database matching; no authentic reference standards were employed. The chemical classes comprised flavonoids, phenolics, lignan glycosides, cardiac glycoside, coumarin, cinnamic acids and limonoids. As previously reported in plant metabolomics, the distribution of metabolites varied significantly among solvents, indicating considerable solvent polarity selectivity [14,15] and, as such, it is imperative that metabolomic studies are conducted using different solvents for comprehensive coverage. Catechin, caffeic acid, glucosyringic acid, hydroquinone-o-glycopyranoside, quinic acid derivatives, and lignan glycosides were among the highly polar phenolic chemicals that predominated in the aqueous extract, reflecting the strong affinity of water for hydrophilic phytochemicals. These chemicals are known to be extremely soluble in water due to glycosylation and hydroxylation patterns [16].

Ethanol yielded the widest chemical diversity of both polar and moderately nonpolar compounds. These included flavonoid glycosides including kaempferol-3-O-rutinoside, quercetin-3-O-rutinoside, cardiac glycoside (neriifolin), and quinone. Ethyl acetate extracts were rich in semipolar flavonoids, and diarylheptanoids such as gingerol, reflecting the affinity of semipolar solvents for these compounds. This fraction showed the highest abundance in ethyl acetate, which preferentially extracts mid-range hydrophobic metabolites [17]. Methanol extracted a broad range of medium-polarity phytochemicals, including phenolic glycosides, flavonoids, coumarins, and limonoids, reflecting its effectiveness in solubilizing both polar and moderately hydrophobic metabolites. This shows methanol’s efficiency in extracting both polar and moderately nonpolar bioactive constituents. Methanol is a widely used solvent for phenolic extraction due to its strong hydrogen-bonding capacity [18] which explains its efficiency in recovering complex bioactive metabolites. Although methanol, ethanol, and ethyl acetate exhibit overlapping polarity ranges, their differential hydrogen-bonding capacity and protic/aprotic characteristics resulted in partial selectivity toward distinct metabolite subclasses. Methanol extracted the broadest range of polar and mid-polar secondary metabolites, including flavonoid glycosides and phenolic acids, whereas ethyl acetate favored less polar aglycones.

### 2.2. Distribution of Antifungal, Antioxidant, and Antimicrobial Metabolites

Table 2 lists metabolites with previously reported antifungal, antioxidant, and/or antibacterial activity in other systems. These molecules were distributed across all four solvent extracts, although the greatest antifungal and antioxidant profiles were seen in the water and methanol extracts, while antimicrobial metabolites were most concentrated in the water, ethanol, and methanol extracts. Antifungal and antioxidant metabolites included flavonoid-3-O-glycosides (methanol), catechins (all solvents), and hydroquinone (water, ethanol and methanol). Antimicrobial metabolites included catechins (all solvents), gingerols (all solvents), limonoids (methanol), and quinone/hydroquinone (water, ethanol and methanol). Methanol extract contained flavonoids, which have been shown to have antibacterial properties that disrupt membranes and cycle redox [19]. Limonoids have strong antifungal and antibacterial characteristics, including activity against *Aspergillus* species [20,21].

### 2.3. Inhibition of Aflatoxin by T. dregeana in Cereal Grains

**Figure 1:**
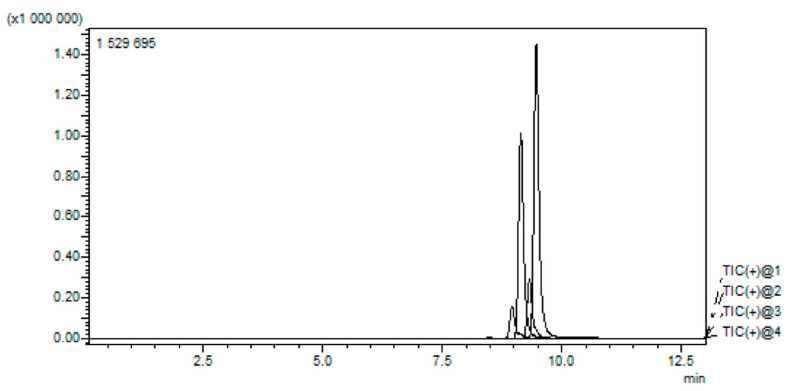
Chromatograms of target analytes (AFB_1_, AFB_2_, AFG_1_, and AFG_2_) obtained by MRM in ESI-positive ionization mode.

**Figure 2:**
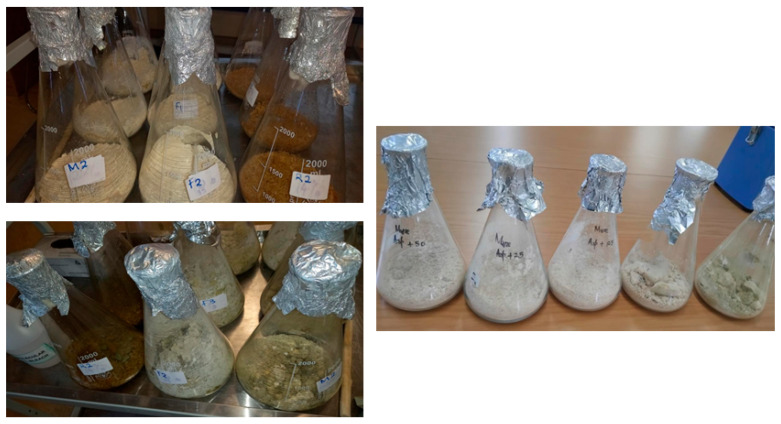
Feed inoculated with *A. flavus* before and after aflatoxin production.

Figure 1 and Figure 2 below show the chromatograph output of the aflatoxin B_1_, B_2_, G_1_, and G_2_ standards and the production of aflatoxin in different feed matrices before inoculation and after 21 days of production, respectively.

Aflatoxin concentrations differed significantly among treatments and feed matrices (*p* < 0.05), Table 3. Significant reductions in aflatoxin levels (µg/kg) were observed only for the methanolic extract, while the water, ethanol, and ethyl acetate extracts did not differ significantly from the untreated control. A supplementary table has been added to present aflatoxin data for all extracts, including those showing no inhibitory effect. The *A. flavus*-inoculated group exhibited higher levels of aflatoxin in all feed matrices; the most contaminated matrix was maize (673.32 µg/kg), followed by rice (448.08 µg/kg) and flour (205.70 µg/kg). This data demonstrated active aflatoxin production after fungal inoculation since its levels were substantially greater than in the uninoculated control. The aflatoxin level in the control flour sample was 102.15 µg/kg. This value remained high after *A. flavus* inoculation (205.70 µg/kg), indicating persistent contamination. Tenazole reduced aflatoxin to 136.66 µg/kg, while the 50 µg/mL extract of *T. dregeana bark* reduced its level to 143.89 µg/kg. Lower concentrations of the extract (12.5 and 25 µg/mL) also showed inhibitory effects, reducing the levels to 159.32 µg/kg and 156.88 µg/kg, respectively. However, control maize had minimal aflatoxin contamination (34.38 µg/kg). Inoculation with *A. flavus* dramatically increased this to 673.32 µg/kg. Treatment with 50 µg/mL *T. dregeana* extract significantly reduced the aflatoxin level to 230.39 µg/kg, lower than Tenazole (445.51 µg/kg). The 25 µg/mL extract yielded 400.02 µg/kg, and the 12.5 µg/mL extract achieved 479.70 µg/kg, all lower than the *A. flavus*-only group. Rice samples inoculated with *A. flavus* showed a sharp rise in aflatoxin concentration from 84.90 µg/kg in the control to 448.08 µg/kg. Tenazole reduced this to 107.13 µg/kg. The 50 µg/mL *T. dregeana* extract similarly reduced the level to 129.93 µg/kg. Lower concentrations (25 µg/mL and 12.5 µg/mL) also reduced aflatoxin levels but remained significantly higher at 220.68 µg/kg and 344.94 µg/kg, respectively.

## 3. Discussion

Solvent-polarity theory, which states that hydrophilic solvents enrich phenolic compounds and glycosides while organic solvents extract terpenoids, lipids, and sterols, is consistent with the different extraction patterns observed across water, ethanol, ethyl acetate, and methanol [14]. The LC-MS profiling of *T. dregeana* extracts demonstrated that solvent polarity critically influences the qualitative composition of phytochemicals, reflecting both the chemical nature of the plant metabolites and their solubility characteristics. Polar solvents such as water and methanol preferentially extracted highly polar phenolic acids and glycosylated flavonoids, consistent with the literature showing that the solubility of phenolic compounds increases with solvent polarity due to hydrogen bonding and dipole interactions with hydroxyl groups on these molecules. Water extracts in this study were enriched in catechin, glucosyringic acid, and caffeic acid, aligning with reports that polar solvents often yield higher levels of glycosylated and hydroxylated phenolics compared to less polar extracts. Similarly, methanol extracts exhibited a broad spectrum of compounds, including flavonoids, phenolic glycosides, and coumarins, supporting prior findings that methanol’s intermediate polarity efficiently dissolves both polar and moderately nonpolar plant metabolites.

The polar solvents extract high quantities of flavonoids, catechins, phenolic acids, and glycosides, which account for their significant antioxidant properties. Phenolic chemicals neutralize free radicals through hydrogen donation and metal chelation, helping to reduce oxidative stress [35]. Antimicrobial metabolites were detected in both hydrophilic and lipophilic fractions, indicating various mechanisms of action. According to Álvarez-Martínez [36], phenolic antimicrobials such as catechins work by disrupting cell walls, inhibiting enzymes, and denaturing proteins. The widespread presence of quinone/hydroquinone suggests that they may be key chemotaxonomic indicators of *T. dregeana* with broad antibacterial applications.

Flavonoids, limonoids, quinones, and catechins have been shown to suppress *A. flavus* growth and aflatoxin biosynthesis [37]. These compounds were found in the methanol extract used in the study. The current work is the first to evaluate the metabolomic content of *T. dregeana* bark extracts and their inhibitory effects on *A. flavus* growth and aflatoxin production across maize, rice, and flour matrices.

The suppression of aflatoxin in maize, rice, and flour reveals that *T. dregeana* bark extract works in a variety of nutritional compositions. The methanol extract exhibited the strongest inhibition, most likely due to its balanced profiles of phenolic antioxidants flavonoid glycosides, phenolic acids and membrane-active metabolites (limonoids, quinone lipids) with known antifungal properties. The overall pattern indicates that aflatoxin reduction levels following treatment with the methanolic extract may be described by synergistic interactions between metabolite classes rather than a single dominant molecule and may be associated with the presence of phytochemical classes previously reported to exhibit antifungal or antimycotoxigenic activity in other systems. This is consistent with the emerging literature demonstrating that plant extracts rich in polyphenols may suppress aflatoxin production through multiple putative mechanisms described in the literature [21,38]. Coumarins such as scopoletin, detected in methanol extracts, have been reported to inhibit fungal sporulation and secondary metabolite production. The broad-spectrum bioactivity found across food matrices suggests that *T. dregeana* bark extracts could be used as natural preservatives to prevent *A. flavus* contamination and aflatoxin production in stored grains and animal feeds. The discovery of flavonoid glycosides and limonoids compounds previously used in plant-derived antifungal formulations confirms the viability of converting these extracts into marketable antimycotoxin medicines.

## 4. Materials and Methods

### 4.1. Plant Material and Extraction

The *T. dregeana* bark used in this study was harvested in KwaZulu-Natal and verified by the Botany Department of North-West University, and a voucher specimen was deposited at S.D. Phalatse herbarium (UNWH) with voucher number Jobe-1 (2026) for future reference. The bark of *T. dregeana* was washed under tap water and rinsed with distilled water before being chopped and air-dried at room temperature to avoid volatile compounds degrading. After drying, it was ground into powder, and 5 g was extracted with 100 mL [39] of either methanol, ethanol, ethyl acetate, or water. The extract was kept for 24 h in an orbital shaker at 150 rpm at room temperature and filtered through 150 mm Whatman filter paper (Lasec^®^ International (Pty) Ltd., Cape Town, South Africa). Two milliliters of the filtrate was filtered through a 0.22 µm syringe filter (Labotec (Pty) Ltd., Johannesburg, South Africa) into amber vials for metabolomic analysis.

#### 4.1.1. Chromatographic Separation and Mass Spectral Analysis

A liquid chromatography–quadrupole time-of-flight tandem mass spectrometer (LC-MS-9030 q-TOF, Shimadzu Corporation, Kyoto, Japan) was used to evaluate the extracts from the *T. dregeana* sample. An Evosphere (Fortis Technologies Ltd., Cheshire, UK) C_18_ column (100 × 2.1 mm, 1.7 µm) kept at 55 °C was used to separate secondary metabolites. Using binary mobile phase gradient elution at a flow rate of 0.3 mL/min^−1^, the bioactive compounds in the *T. dregeana* extracts were separated after a volume of 5 µL was injected into the device. Formic acid (0.1%: *v*/*v*) in ultra-pure water and methanol were used as mobile phases A and B, respectively. The mobile phase composition was maintained at 5% for mobile phase B from 0 to 3 min and then kept at that level for 8 min. After 8 min, B’s proportion increased to 40%, and between 23 and 25 min, it reached 95%. After 27 min, the gradient was changed to 5% mobile phase B, and it remained at this composition until 30 min, re-equilibrating the column for the next run. Mass spectral analysis was conducted using a q-TOF high-resolution mass spectrometer with an electrospray interface (ESI) functioning in negative ionization mode.

Nebulization and dry gas flow of 3 L min^−1^, heat block temperature of 400 °C, DL temperature of 280 °C, detector voltage of 1.8 kV, interface voltage of 4.0 kV, interface temperature of 300 °C, and light tube temperature of 42 °C were the parameters of the mass spectrometer. High mass accuracy was monitored using sodium iodide as a calibration solution. MS_1_ and MS_2_ were produced simultaneously via data-dependent acquisition for every ion with an intensity threshold above 3000 and *m*/*z* values between 100 and 1000 Da. Argon was used as a collision gas to generate MS_2_ data, with a collision energy of 30 eV.

#### 4.1.2. Molecular Identification

The Shimadzu LCMS-9030 q-TOF’s raw data was converted to mzML format and processed with Sirius (v6.1) to predict chemical formulas and annotate compounds. The detected molecules were compared against the KNApSAcK natural products database (https://www.knapsackfamily.com/knapsack_core/top.php; accessed on 26 January 2026), and their accurate masses, retention times, fragment ions, and compound names were recorded.

### 4.2. Feed Inoculation and Treatment

The *Aspergillus* strain (MG659624) was revived on PDA (Merck KGaA, Darmstadt, Germany) media that was prepared following the manufacturer’s protocol. Briefly, 200 g of each feed sample was weighed into a flask and autoclaved at 121 °C to remove any debris, and the feed was placed in a 100 °C oven for 24 h to achieve a 0% moisture content [40]. Treatments were assigned to cereal matrices using a randomized complete design, with extract treatments and controls randomly allocated to experimental units using a random number generator prior to incubation. This procedure was implemented to minimize allocation bias and ensure comparability among treatment groups. The experiment comprised 3 treatments (control, *A. flavus*-only, and *A. flavus* + treatments), 3 matrices, 3 extract concentrations 12.5, 25, and 50 mg/mL, and tenazole, each in triplicate, giving a total of 24 experimental units. The fungal spore suspension was used to inoculate the feed, where 2 mL of 2 × 10^−2^ was added to the feed, and respective treatments of either *T. dregeana* extract or tenazole and distilled water were used to achieve a moisture content of 15%. The 15% moisture levels of the cereal matrices were adjusted according to the established feed inoculation protocols to provide optimal conditions for fungal growth and aflatoxin production [41]. The organic extracts were dried after extraction and redissolved in water before use to avoid the solvent being an antimicrobial variable. The feed flasks were incubated at 30 °C to allow fungal growth and mycotoxin production, while constantly monitoring and mixing on a weekly basis for 21 days. Aflatoxin inhibition experiments were independently repeated three times, with each experimental run conducted on separate occasions using freshly prepared fungal inoculum, feed matrix, and extract solutions. Within each independent experiment, all treatments were performed in triplicate. On day 21, the samples were placed in a Ziplock bag and stored at 20 °C freezer for further analysis of aflatoxin quantification (HPLC and LC-MS).

### 4.3. Quantification of Aflatoxins Using LC-QqQ-MS

#### 4.3.1. Sample Preparation

A total of 50 g of the respective homogenized feed sample was used for aflatoxin detection while blinding the treatment identity to minimize bias and ensure objective measurement of mycotoxin level. The aflatoxin extraction was performed using the Easi Extract^®^ aflatoxin immunoaffinity columns (^®^Biopharm Rhone Ltd., Glasgow, UK) according to the manufacturer’s protocol. Briefly, 100 mL of 80% methanol was added to 50 g of the sample and 5 g of sodium chloride and allowed to mix thoroughly for 20 min in a blender at high speed. The sample was filtered through a Whatman no. 4 filter paper into clean beakers. In total, 2 mL of the filtered extract was diluted with 14 mL phosphate-buffered saline (PBS) solution, then allowed to pass through the column at a flow rate of 1 drop/second to enable capture of the aflatoxins by the antibody. The column was further washed by passing through 20 mL PBS to remove residual liquid. Then, 2 mL of 100% methanol was used to elute the toxins from the column, and samples were further filtered using micro-pore syringe filters (0.22 μm) into an amber vial.

Aflatoxin detection and quantification were performed using HPLC (Shimadzu FCV-20H2) (Shimadzu Corporation, Kyoto, Japan) with operation conditions as given in the KOBRA^®^ cell (Shimadzu Corporation, Kyoto, Japan) instruction manual. In total, 10 µL was injected into the HPLC system, and calibration curves for each aflatoxin were constructed using total aflatoxin standard (B_1_, B_2_, G_1_, and G_2_) (Trilogy, Washington, DC, USA) solutions. The aflatoxin concentration of the samples was calculated by measuring the area of the peak and then interpolating from the standard curve.

#### 4.3.2. LC-MS/MS Analysis

Briefly, an 8050 LC-MS/MS triple quadrupole System (Shimadzu Corporation, Kyoto, Japan) equipped with a Turbo Ion Spray electrospray ionization (ESI) source and a 1100 Series HPLC System was used. Chromatographic separation of the conjugates was performed using a Shimadzu C_18_ column (150 × 2.1 mm, 5 μm) (Shimadzu Corporation, Kyoto, Japan) with mobile phases A (10 mM ammonium acetate in water) and B (2% acetic acid in methanol). The flow rate was 0.25 mL/min with an injection volume of 10 µL for all standards and samples. The ESI-MS/MS source temperature was 550 °C, in multiple reaction monitoring (MRM) mode, in both positive and negative polarities, in two separate chromatographic runs per sample by scanning two fragmentation reactions per analyte. Further MS parameters were as follows: curtain gas 10 psi (69 kPa of max. 99.5% nitrogen); ion source gas 1 (sheath gas) 50 psi (345 kPa of nitrogen); ion source gas 2 (drying gas) 50 psi (345 kPa of nitrogen); ion spray voltage −4000 V and +4000 V, respectively and collision-activated dissociation gas (nitrogen) high. For the aflatoxin, the running conditions were as shown in Table 4.

### 4.4. Statistical Analysis

Quantitative data were analyzed using one-way ANOVA in SAS 9.4, for each sample type (rice, maize, and flour) and followed by Tukey’s HSD post hoc test. The results are presented as the mean ± standard deviation.

## 5. Conclusions

The integrated metabolomic and antifungal investigation shows that *T. dregeana* bark extracts include a diverse range of phenolics, terpenoids, limonoids, saponins, and quinone lipids that work together to suppress *A. flavus* growth and aflatoxin production. The substantial association between metabolite composition and bioactivity across solvents emphasizes the importance of specific chemical classes in inhibiting aflatoxin production. These findings support the continued development of *T. dregeana* extracts as natural antifungal agents for food and feed safety. However, the findings are limited to crude-extract-level observations, with significant aflatoxin inhibition observed only for the methanolic extract under controlled experimental conditions. Limitations include the absence of fungal biomass measurements, mechanistic validation, toxicity assessment, and regulatory evaluation. Therefore, to advance toward practical application, future studies are required to isolate and characterize the active compounds through fractionation. Comprehensive toxicity testing is essential to ensure safety for animal and human consumption, and any potential use must undergo rigorous regulatory assessment to meet feed additive standards. Collectively, these steps are critical to translating the observed bioactivity into safe, effective, and commercially viable interventions for feed and food safety.

## Figures and Tables

**Table 1:** Chemical composition of *T. dregeana bark* extract in different extraction solvents, water, ethanol, ethyl acetate, and methanol using negative ionization.

Compound Class	Compound Name	Compound Formula	Retention Time (min)	Precursor Ion (*m*/*z*)	Fragment Ions	Water	Ethanol	Ethyl Acetate	Methanol
Flavonoid	Catechin	C_15_H_14_O_6_	5.86	289.0714	151, 123, 109	x	x	x	x
Flavonoid	Procyanidin B2	C_30_H_26_O_12_	7.34	577.1355	289, 125				x
Flavonoid	Kaempferol-3-O-rutinoside	C_30_H_26_O_13_	11.72	593.1308	447, 285, 284		x	x	x
Flavonoid	Quercetin-3-O-rutinoside	C_30_H_26_O_14_	10.95	609.1258	463, 301, 300		x		x
Flavonoid	Quercetin-3-O-xylosylglucoside	C_26_H_28_O_16_	8.73	595.1311	301, 300				x
Flavonoid	Quercetin-3-O-glucoside	C_21_H_20_O_12_	9.25	463.0888	301, 300				x
Diarylheptanoid	Gingerol	C_17_H_26_O_4_	15.32	293.1754	221, 220, 205, 192	x	x	x	x
Cardiac glycoside	Neriifolin	C_30_H_46_O_8_	21.76	533.3119	417, 399, 355, 331, 145		x	x	x
Lignan glycoside	Lyoniresinol glucopyranoside	C_28_H_38_O_13_	8.51	581.2245	419, 404, 373, 233	x	x		x
Phenolic glycoside	Hydroquinone-O-glucopyranoside	C_12_H_16_O_7_	7.72	271.0822	139	x	x		x
Phenolic acid	Glucosyringic acid	C_15_H_20_O_10_	8.64	359.0985	153, 121	x			x
Cinnamic acid	Caffeic acid	C_9_H_8_O_4_	6.59	179.0348	135	x			x
Chlorogenic acid	3-coumaroylquinic acid	C_16_H_18_O_8_	7.67	337.0922	119, 111	x		x	
Coumarin	Scopoletin	C_12_H_16_O_7_	8.30	191.0347	148, 120, 104	x	x	x	x
Limonoid	12-O-deacetyltrichilin H	C_34_H_44_O_13_	18.98	659.2780	131				x

**Table 2:** Compounds with both antioxidant and antimicrobial properties across all extracts.

Compound Class	Compound Name	Bioactivity (Extract Level)	Water	Ethanol	Ethyl Acetate	Methanol	References
Flavonoid-3-O-glycosides	Quercetin-3-O-glycoside	antioxidant, antifungal				x	[22,23]
Lignan glycosides	Lyoniresinol	antioxidant, antimicrobial, antifungal	x			x	[24,25]
Phenols	Chlorogenic acid	antioxidant, antifungal	x			x	[26,27,28]
Flavonoid	Catechins	antioxidant, antimicrobial, antifungal	x	x	x	x	[29]
Cinnamic acid	Caffeic acid	antioxidant, antifungal		x			[30]
Phenolic glycosides	Quinone & hydroquinone	antimicrobial, antifungal	x	x		x	[31]
Diarylheptanoid	Gingerols	antioxidant, antimicrobial, antifungal	x		x	x	[32,33]
Limonoids	12-O-deacetyltrichilin H	antimicrobial, antifungal				x	[20,34]

**Table 3:** Effect of *T. dregeana* methanol extract on aflatoxin production (in µg/kg) in different feed matrices (flour, maize, and rice) (mean ± SD, µg/kg aflatoxin; *n* = 3).

Treatment	Flour (µg/kg)	Maize (µg/kg)	Rice (µg/kg)
Control	102.15 ^a^ ± 16.55	34.38 ^e^ ± 0.75	84.90 ^f^ ± 9.26
*A. flavus*	205.70 ^b^ ± 25.49	673.32 ^a^ ± 13.54	448.08 ^a^ ± 2.06
Tenazole	136.66 ^d^ ± 0.47	445.51 ^c^ ± 4.92	107.13 ^e^ ± 0.78
*A. flavus* + 12.5 µg/mL	159.32 ^c^ ± 2.79	479.20 ^b^ ± 2.63	344.94 ^b^ ± 16.36
*A. flavus* + 25 µg/mL	156.88 ^c^ ± 15.63	400.02 ^c^ ± 0.41	220.68 ^c^ ± 22.85
*A. flavus* + 50 µg/mL	143.89 ^d^ ± 16.3	230.39 ^d^ ± 0.15	129.93 ^d^ ± 7.29

Groups with the same letter are not significantly different (Tukey HSD, *p* < 0.05).

**Table 4:** Retention times and mass precursor for aflatoxins.

Target Compounds	Retention Time (min)	MRM Transition (*m*/*z*)
AFB_1_	9.464	313.10 > 241.10
AFB_2_	9.332	315.10 > 259.10
AFG_1_	8.684	329.10 > 243.10
AFG_2_	8.971	331.10 > 245.10

## Data Availability

The original contributions presented in this study are included in the article. Further inquiries can be directed to the corresponding author.
